# 

**Published:** 1880-07

**Authors:** 


### NOTICE.

National Dental Association.—The next annual meeting of the National Dental Association will be held in New York city on Wednesday, August (11th) eleventh at 10 o'clock A. M. Further particulars will be timely given through circulars of committee of arrangements. All are invited.

### EXCURSION

*To the American Dentist Association to be held in Boston, August 3d, 1880.*

For the pleasure and economy of the members of the dental profession, going to the Association, and their friends, a splendid excursion has been arranged under the auspices and guidance of the agent of the Canada Southern Railway, Mr. H. F. Eberts.

The starting points are Detroit, Toledo and Cincinnati. The time and route will be about as follows: Leave Cincinnati at

2:30 P. M.; Detroit and Toled› at about 10 o'clock P. M. on Monday July 26th; arrive at Buffalo next morning, then on to Syracuse; there dine at 12:30 P. M.; from there to Cape Vincent, and there stop over night. At 7 A. M. Wednesday, take the steamer and pass through the Thousand Islands and the Rapids, arriving at Montreal at 6 P. M. Thence by steamer to Quebec, arriving there Thursday at 6 A. M.. The day will be spent in visiting all matters of interest in this quaint old city. Leave this place at 6 P. M. and arrive at Montreal next morning at 6 o'clock. Friday will be devoted to visiting all the points of interest in this city. Leaving Montreal Saturday at 8 P. M. for Vermont and White Mountains. Sabbath will be spent at the Profile House, a most delightful place in Franconia Notch. On Monday morning at 7:45, leave for Mount Washington, upon arrival here the party will ascend to the top, take dinner on the summit; here most of the day will be spent. Returning, reach the Fabyan House in time for supper.

All the expenses of the party up to this point is paid by the agent.

The party or any portion of it can leave for Boston on Monday night and arrive next morning in time for the meeting, or they may linger for several days and then proceed. The payment of expenses, except transportation by the agent, ceases upon leaving the Fabyan House.

The return from Boston will be by Old Colony R. R. and Fall River steamers to New York. By day steamer, Hudson River, to Albany. thence to Buffalo, via. New York Central, then by Canada Southern, home.

The ticket for this entire trip, all expenses paid by agent to Fabyan House as indicated above will be from Detroit or Toledo, $60; from Cincinnati, $65. The ticket will be good until September 25th.

An excursion on the same route will start July 19th, this will accomodate some who wish to start eariler than the 26th. To those wishing fuller particulars circulars will be sent on application to the editor of the Register or to Mr. H. F. Eberts, Detroit, Michigan.

### CONTENTS.

### COMMUNICATIONS.

Dental Education.... 293
What Do You Do it For.... 299
Address.... 304
Pathology and Semiology.... 310
Cylinder Filling.... 316
Tardy Eruption of the Teeth.... 318
Clinic Reports.... 320
Scientific News.... 321
Correspondence.... 325

### EDITORIAL.

An Important Movement.... 327
Call for a Mass Convention.... 328
Dennett's Dental Naboli.... 331
A New Appliance.... 335
Bibliographical.... 336
Obituary.... 336
Excursion.... 337

---

### THE DENTAL REGISTER,

A MONTHLY MAGAZINE DEVOTED TO THE INTERESTS OF

**THE DENTAL PROFESSION.**

*TERMS REDUCED TO $2.50 PER YEAR. SINGLE COPIES 25C.*

EDITED BY PROF. J. TAFT, D.D.S.

AND

**Published by SPENCER, CROCKER & CO., Ohio Dental Depot.**

The volume commences in January and closes in December.

The REGISTER being issued monthly is always fresh, therefore Indispensable to the practitioner who desires to keep posted up with the new inventions and improvements which are daily developed. Neither expense nor effort will be spared to give value and variety to its contents. Articles will be illustrated with well executed engravings whenever they will add to the interest or assist to explanation.

Its extensive circulation and frequency of issue make it one of the BEST DENTAL ADVERTISING MEDIUMS in the United States.

All subscriptions must begin with January or July.

Local or special notices, five lines or less, will be inserted for $3 each insertion; over five lines, 50 cts. per line.

All advertising bills payable in advance, or at furthest, after the issue of the first number containing the advertisement. All transient advertisements to be paid for in advance.

☞CONTRIBUTIONS SOLICITED.

Exclusively Editorial Communications should be addressed to Editor.

The latest number of the REGISTER may be ordered with or without oth-[illegible] goods at any time, and all letters should be addressed to

***SPENCER, CKOCKER & CO., Ohio Dental Depot, Cincinrati, O.***

